# No effects of transcranial DLPFC stimulation on implicit task sequence learning and consolidation

**DOI:** 10.1038/s41598-017-10128-0

**Published:** 2017-08-29

**Authors:** Branislav Savic, Dario Cazzoli, René Müri, Beat Meier

**Affiliations:** 1 Institute of Psychology and Center for Cognition, Learning, and Memory, University of Bern, Bern, Switzerland; 2 Bern University Hospital, and Center for Cognition, Learning, and Memory, Bern, Switzerland; 3 Bern University Hospital, and ARTORG Center for Biomedical Engineering Research, Bern, Switzerland

## Abstract

Neurostimulation of the dorsolateral prefrontal cortex (DLPFC) can modulate performance in cognitive tasks. In a recent study, however, transcranial direct current stimulation (tDCS) of the DLPFC did not affect implicit task sequence learning and consolidation in a paradigm that involved bimanual responses. Because bimanual performance increases the coupling between homologous cortical areas of the hemispheres and left and right DLPFC were stimulated separately the null findings may have been due to the bimanual setup. The aim of the present study was to test the effect of neuro-stimulation on sequence learning in a uni-manual setup. For this purpose two experiments were conducted. In Experiment 1, the DLPFC was stimulated with tDCS. In Experiment 2 the DLPFC was stimulated with transcranial magnetic stimulation (TMS). In both experiments, consolidation was measured 24 hours later. The results showed that sequence learning was present in all conditions and sessions, but it was not influenced by stimulation. Likewise, consolidation of sequence learning was robust across sessions, but it was not influenced by stimulation. These results replicate and extend previous findings. They indicate that established tDCS and TMS protocols on the DLPFC do not influence implicit task sequence learning and consolidation.

## Introduction

Stimulation of the prefrontal cortex with electric and magnetic fields can be used to influence several different cognitive abilities including learning and memory^1–3^. However, since these effects are variable further understanding of prefrontal cortex stimulation is needed^4, 5^. Indeed, in a recent study, we have found no effects of dorsolateral prefrontal cortex (DLPFC) stimulation in a task in which patients with DLPFC lesions were strongly impaired^6^. In that study, implicit task sequence learning was investigated with a paradigm that involved bimanual responses. Since tasks performed with two hands increase the coupling between homologous cortical areas of the hemispheres^7–9^, and in that study the left and right DLPFCs were stimulated one at a time, the null results could have been provoked by the use of two hands. That is, the increased coupling between homologous cortical areas of the hemispheres may have obscured the specific left vs. right hemisphere effects of tDCS. Therefore, in this study, we focused on uni-manual task performance to increase hemisphere specific processing and, accordingly, the stimulation effects.

Many studies have demonstrated that regular sequences of events, in particular sequences of motor responses and perceptual stimuli, can be learned incidentally. This kind of learning results in knowledge difficult to express consciously, that is, implicit sequence learning^10^. Only recently, with the development of the task sequence learning paradigm (TSL), it was shown that incidental learning is also possible for sequences of tasks, which do not involve motor response and stimulus sequences^11–13^. In a typical TSL paradigm, participants see a series of stimuli, for example digits or letters, for which they have to perform a particular decision task. When a digit appears, they have to decide if it is smaller or bigger than five. When a letter appears they have to decide if it is a vowel or a consonant. When the digit or the letter is green, participants have to give a left hand response for digits smaller than five and vowels, and a right hand response for digits bigger than five and consonants. When the digit or the letter is presented in red, they have to use the opposite key mapping. Unbeknownst to participants, the order of tasks (digit vs. letter task) and of response mappings (green vs. red) follow a sequence, which is presented during several blocks of trials (i.e., sequenced blocks). The results show that throughout reaction times (RTs) decrease. However, when the sequenced order is changed to random, RTs increase and this increase can be taken as indirect evidence for sequence-specific learning.

If sequence-specific memory traces become stable with time passing by, then consolidation has occurred^14^. Typically, consolidation is measured by repeating a task in two sessions separated by a period of time in which participants do not practice the task^15^. Previous studies have shown that learning remained stable or even improved across sessions^16–22^. In line with these results, our previous study^6^ showed that memory traces of task sequences were stable across sessions.

Regarding the neural basis of implicit task sequence learning, the DLPFC seems critical as patients with DLPFC lesions do not exhibit task sequence learning^23^. This is in line with the fact that task switching, which is involved in the TSL, also requires proper DLPFC functioning^24–26^. Typically, RTs increase in trials in which the task is switched compared to trials in which the task is repeated, that is, switching comes at costs^27, 28^. Here, these costs can be taken as a control parameter to evaluate whether or not the DLPFC was successfully stimulated.

The aim of the present study was to influence sequence-specific learning in the TSL performed uni-manually by stimulating the DLPFC. In Experiment 1, participants received transcranial direct current stimulation (tDCS) above the left or the right DLPFC during the TSL. The tDCS protocol of Ohn *et al*.^29^ was adopted since it seems to influence a memory task for up to 30 minutes. To evaluate the impact of tDCS on consolidation 24 hours later, participants were tested again. Because tDCS has a low precision and many recent results highlighted the variability of its effects^30–34^, in Experiment 2 the DLPFC was stimulated with transcranial magnetic stimulation (TMS) which has a higher precision^cf.^ ^35–37^. In order to increase the chances of influencing sequence-specific learning with TMS, two different stimulation protocols were used. For the left DLPFC, there is robust evidence that the low frequency repetitive (rTMS) protocol of Mottaghy *et al*.^38^ diminishes implicit sequence learning and memory^38–40^. Thus, rTMS was used to stimulate the left DLPFC. More generally, several studies have shown that continuous theta burst stimulation (cTBS) can reduce cortical excitability. Thus, we used an established cTBS protocol of Nyffeler *et al*.^41^ to stimulate the right DLPFC.

As previous results showed that left hemisphere stimulation modulates memory tasks, we expected that left DLPFC stimulation would influence TSL performance^42–44^. Moreover, because in the TSL different types of information are integrated in the same task (i.e., the information about digits and letters and information about response mapping) and the right hemisphere seems involved in information integration, right DLPFC stimulation was as well expected to influence sequence-specific learning^45, 46^. Specifically, in Experiment 1, we expected that anodal stimulation would enhance learning and that cathodal tDCS would reduce learning, In Experiment 2, we expected TMS to reduce learning.

## Results

### Experiment 1

#### Sequence learning and consolidation

**Figure 1:**
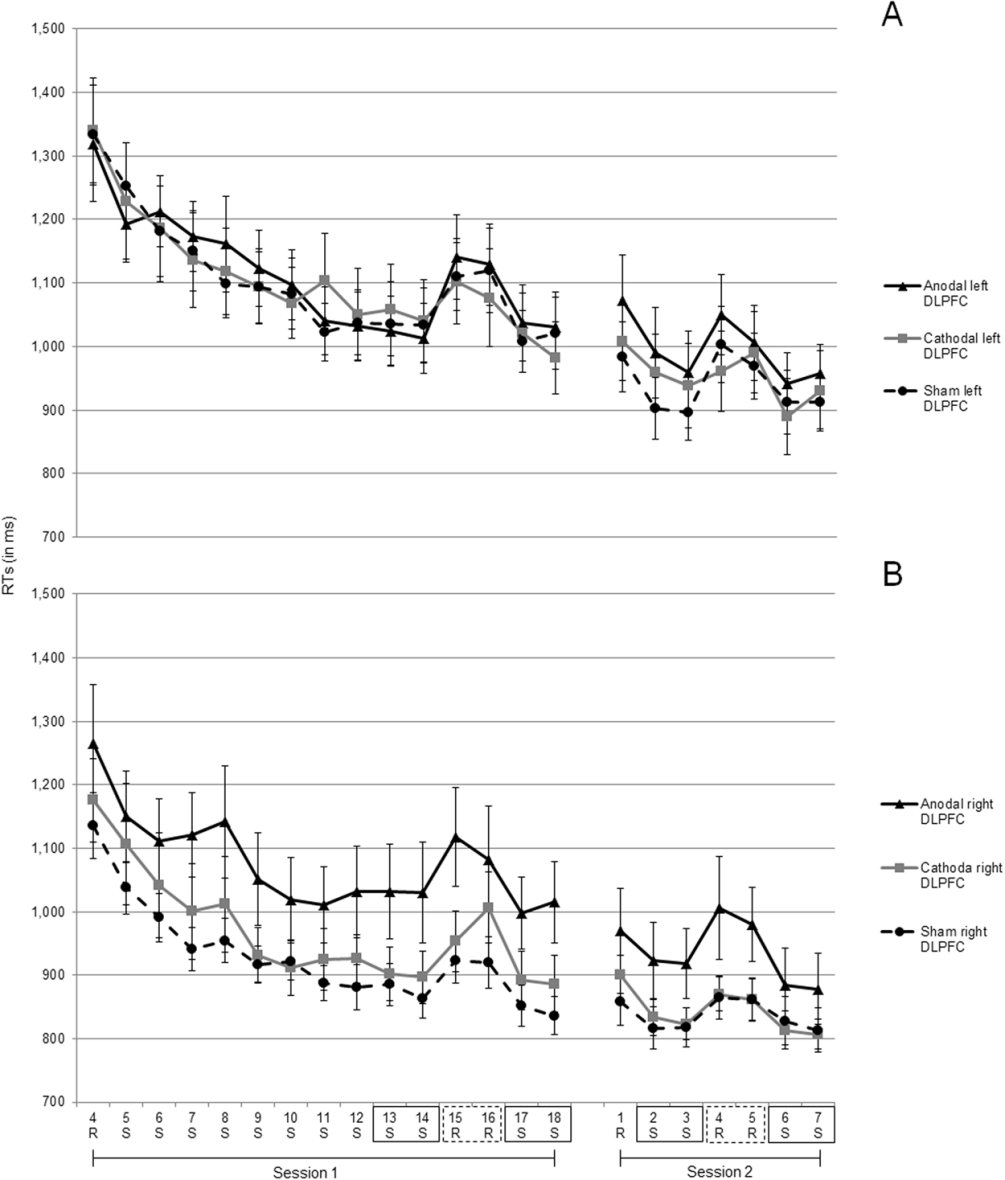
RT trajectories across blocks, sessions, and type of stimulation (anodal, cathodal, and sham) for Experiment 1, separately for (**A**) left DLPFC and (**B**): right DLPFC. “R” indicates a pseudorandom block; “S” indicates a sequenced block. The RT difference between blocks highlighted by the black rectangles and the blocks highlighted by the dashed rectangles were used to calculate disruption scores indicative of sequence learning (i.e., 13, 14, 17, 18 vs. 15, 16 for Session 1, and 2, 3, 6, 7 vs. 4, 5 in Session 2, respectively). Bars represent standard errors.

**Figure 2:**
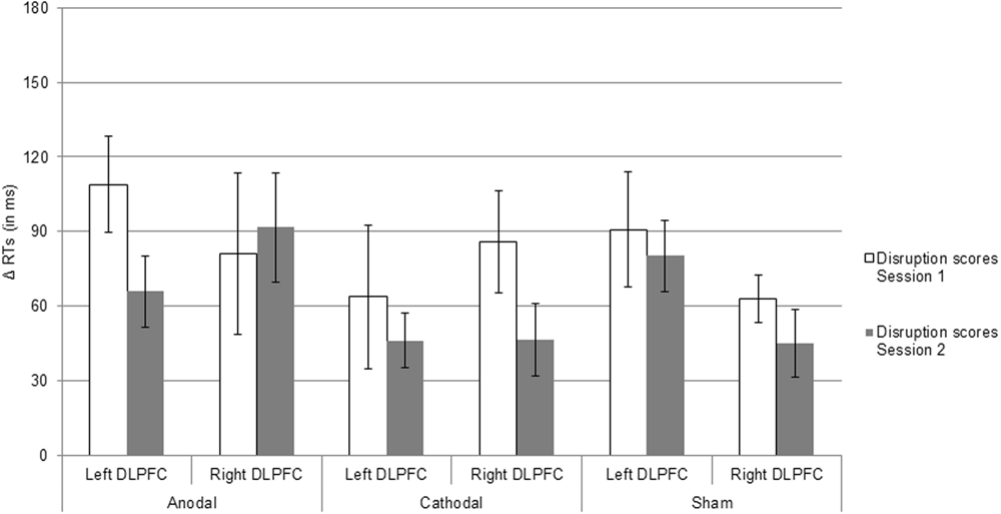
Disruption scores for Experiment 1, separately for Session 1 and Session 2, and each experimental condition, respectively. Bars represent standard errors.

The RT results in milliseconds (ms) are depicted in Fig. 1. For Session 1, to investigate the influence of tDCS on sequence-specific learning an analysis of variance (ANOVA) was conducted with blocks (mean RTs of pseudorandom blocks 15 and 16 vs. mean RTs of sequenced blocks 13, 14, 17, and 18) as within subject factor, and stimulation type (anodal, cathodal, sham) and hemisphere (left DLPFC, right DLPFC) as between subjects factors. The ANOVA showed a significant main effect of block, *F* (1, 77) = 73.00, *p* < 0.001, ƞ^2^ = 0.48, indicating that in all conditions RTs increased when the sequence was switched to pseudorandom (i.e., sequenced blocks = 973 ms, *SE* = 22; pseudorandom blocks = 1055 ms, *SE* = 25). Furthermore, the analysis showed a significant main effect of hemisphere, *F* (1, 77) = 5.57, *p* < 0.05, ƞ^2^ = 0.07, indicating that the mean RTs of blocks 13–18 were larger in the left than in the right DLPFC condition (i.e., left DLPFC = 1054 ms, *SE* = 32; right DLPFC = 948 ms, *SE* = 30). In addition, for the disruption scores (i.e., RT difference between the mean of pseudorandom blocks 15 and 16 and the mean of the surrounding sequenced blocks 13, 14, 17, and 18) of each condition one sample *t*-tests against zero were conducted. The *t*-test for the anodal left DLPFC gave *t* (11) = 5.65, *p* < 0.001, for anodal right DLPFC *t* (13) = 2.48, *p* < 0.05, for cathodal left DLPFC *t* (12) = 2.22, *p* < 0.05, for cathodal right DLPFC *t* (12) = 4.17, *p* < 0.001, for sham left DLPFC *t* (15) = 3.91, *p* < 0.001, and for sham right DLPFC *t* (14) = 6.47, *p* < 0.001 (see Fig. 2), indicating that in all conditions par*t*icipants showed sequence-specific learning.

For Session 2, the mixed three factorial ANOVA for investigating tDCS influences on sequence-specific learning showed a main effect of block, *F* (1, 77) = 96.56, *p* < 0.001, ƞ^2^ = 0.55, indicating that in all conditions RTs increased when the sequenced order was switched to pseudorandom (i.e., sequenced blocks = 887 ms, *SE* = 19; pseudorandom blocks = 950 ms, *SE* = 22). Also, there was a main effect of hemisphere, *F* (1, 77) = 4.77, *p* < 0.05, ƞ^2^ = 0.06, indicating that the mean RTs of blocks 2–7 were larger in the left than in the right DLPFC condition (i.e., left DLPFC = 951 ms, *SE* = 30; right DLPFC = 866 ms, *SE* = 24). For the disruption score (i.e., RT difference between the mean of pseudorandom blocks 4 and 5 and the mean of the surrounding sequenced blocks 2, 3, 6, and 7) one sample *t*-tests against zero of anodal left DLPFC gave *t* (11) = 4.56, *p* < 0.001, for anodal right DLPFC *t* (13) = 4.12, *p* < 0.001, for cathodal left DLPFC *t* (12) = 4.14, *p* < 0.001, for cathodal right DLPFC *t* (12) = 3.17, *p* < 0.01, for sham left DLPFC *t* (15) = 5.64, *p* < 0.001, and for sham right DLPFC *t* (14) = 3.33, *p* < 0.05 (see Fig. 2), indicating that in all conditions par*t*icipants showed sequence-specific learning.

The consolidation results are depicted in Fig. 2. To investigate the influence of tDCS on consolidation of sequence-specific learning, a mixed three factorial ANOVA was conducted with the disruption scores of Session 1 and 2 as within subject factor, and stimulation type (anodal, cathodal, sham) and hemisphere (left DLPFC, right DLPFC) as between subjects factor. The analysis showed no significant effect (*ps* > 0.08), indicating that in all conditions disruption scores did not change across sessions.

#### Switch costs

Switch costs were calculated as the RT difference between trials in which the task was switched and trials in which it was repeated. For Session 1, switch costs were calculated on pseudorandom blocks 15 and 16. For Session 2, switch costs were calculated on pseudorandom blocks 3 and 4. Average switch costs were 120 ms (*SE* = 20) and 109 ms (*SE* = 14) for Session 1 and 2, respectively. T-tests showed that these costs were statistically different from zero, *t* (82) = 5.91, *p* < 0.001, for session 1 and *t* (82) = 7.45, *p* < 0.001, for session 2, respectively. To investigate the influence of tDCS a mixed three factorial ANOVA was conducted with the switch costs of Session 1 and 2 as within subject factor, and stimulation type (anodal, cathodal, sham) and hemisphere (left DLPFC, right DLPFC) as between subjects factor. The analysis showed no significant effect (*ps* > 0.08), indicating that tDCS also did not influence task switching performance. The detailed descriptive results are presented in the Supplementary Figure S1.

The *p*-value of 0.08 refers to a trend for switch costs to be different between anodal, cathodal, and sham conditions. Due to the theoretical and practical implications, we conducted follow-up post-hoc tests which only revealed a marginal significant effect for switch costs being larger in the anodal than in the sham group, *p* = 0.064 (i.e., anodal = 160 ms, *SE* = 35; cathodal = 116 ms, *SE* = 27; sham = 76 ms, *SE* = 17). However, since there were no interactions with factors stimulation type and hemisphere, this marginally significant *p*-value is not relevant to the main question. We would like to emphasize that even if one considers this “borderline” effect of stimulation type as meaningful, it would indicate that despite the possibility that stimulation type affected switch costs, it did not have any effect on sequence learning.

### Experiment 2

#### Sequence learning and consolidation

**Figure 3:**
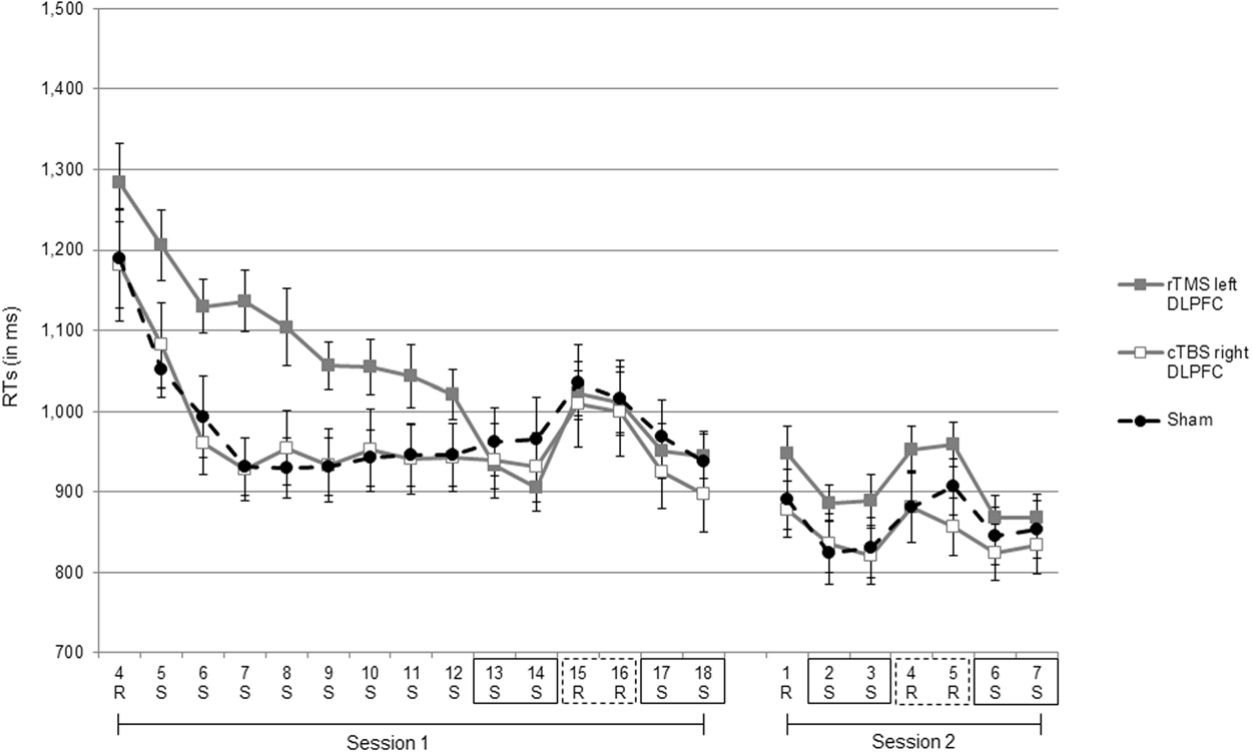
RTs trajectories across blocks, sessions, and type of stimulation (rTMS left DLPFC, cTBS right DLPFC, sham) for Experiment 2. “R” indicates a pseudorandom block; “S” indicates a sequenced block. The RT difference between blocks highlighted by the black rectangles and the blocks highlighted by the dashed rectangles were used to calculate disruption scores indicative of sequence learning (i.e., 13, 14, 17, 18 vs. 15, 16 for Session 1, and 2, 3, 6, 7 vs. 4, 5 in Session 2, respectively). Bars represent standard errors.

**Figure 4:**
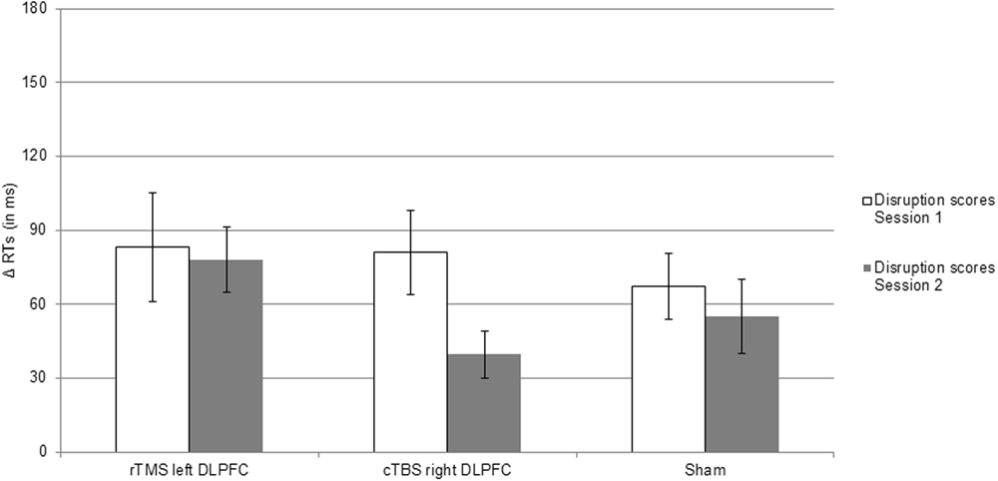
Disruption scores for Experiment 2, separately for Session 1 and Session 2, and each experimental condition, respectively. Bars represent standard errors.

The RT results are depicted in Fig. 3. For Session 1, to investigate whether TMS influenced sequence-specific learning a mixed two factorial ANOVA was conducted, with blocks (mean RT of pseudorandom blocks 15 and 16 vs. mean RT of sequenced blocks 13, 14, 17, and 18) as within subject factor, and stimulation type (rTMS left DLPFC, cTBS right DLPFC, sham) as between subjects factor. The ANOVA showed a significant main effect of block, *F* (1, 45) = 56.93, *p* < 0.001, ƞ^2^ = 0.55, and no other significant main effect or interaction (*p*s > 0.79), indicating that in all conditions RTs increased when the sequence was switched to pseudorandom (i.e., sequenced blocks = 938 ms, *SE* = 22; pseudorandom blocks = 1015 ms, *SE* = 25). For the disruption score one sample *t*-tests against zero of rTMS left DLPFC gave *t* (15) = 3.79, *p* < 0.05, for cTBS right DLPFC *t* (15) = 4.79, *p* < 0.001, and for sham *t* (15) = 5.08, *p* < 0.001 (see Fig. 4), indicating that in all conditions participants showed sequence-specific learning.

For Session 2, the mixed two factorial ANOVA for investigating TMS influence on sequence-specific learning showed a main effect of block, *F* (1, 45) = 62.48, *p* < 0.001, ƞ^2^ = 0.58, indicating that in all conditions RTs increased when the sequenced order was switched to pseudorandom (i.e., sequenced blocks = 848 ms, *SE* = 18; pseudorandom blocks = 906 ms, *SE* = 20). For the disruption score one sample *t*-tests against zero of rTMS left DLPFC gave *t* (15) = 6.01, *p* < 0.001, for cTBS right DLPFC *t* (15) = 4.22, *p* < 0.05, and for sham *t* (15) = 3.70, *p* < 0.05 (see Fig. 4), indicating that in all conditions participants showed sequence-specific learning.

The consolidation results are depicted in Fig. 4. To investigate the influence of TMS on consolidation of sequence-specific learning, a mixed two factorial ANOVA was conducted with the disruption scores of Session 1 and 2 as within subject factor, and stimulation type (rTMS left DLPFC, cTBS right DLPFC, sham) as between subjects factor. The analysis showed no significant effect (*ps* > 0.07), indicating that in all conditions disruption scores did not change across sessions.

#### Switch costs

Average switch costs were 123 ms (*SE* = 17) and 94 ms (*SE* = 14) for Session 1 and 2, respectively. T-tests showed that these costs were statistically different from zero, *t* (47) = 7.09, *p* < 0.001, for session 1 and *t* (47) = 6.67, *p* < 0.001, for session 2, respectively. To investigate the influence of TMS a mixed two factorial ANOVA was conducted with the switch costs of Session 1 and 2 as within subject factor, and stimulation type (rTMS left DLPFC, cTBS right DLPFC, sham) as between subjects factor. The analysis showed no significant effect (*ps* > 0.14), indicating that TMS also did not influence task switching performance. The detailed descriptive results are presented in the Supplementary Figure S2.

## Discussion

Recent results showed that DLPFC tDCS does not influence implicit task sequence learning and consolidation when a task sequence learning paradigm was performed bimanually^6^. As bimanual task performance requires the coupling between homologous cortical regions of the hemispheres and, because the left and the right DLPFC were stimulated one at the time, the null findings could have been provoked by the bimanual set-up of the task. Specifically, the increased coupling between homologous cortical areas of the hemispheres may have obscured the hemisphere-related effects of tDCS. To expand our understanding of DLPFC stimulation, in the present study, we sought to influence implicit task sequence learning and consolidation in a uni-manual set-up. For this purpose the DLPFC was stimulated with tDCS and TMS. In line with previous findings the results showed that sequence-specific learning was present in all conditions and sessions. This result further supports that implicit task sequence learning can be measured reliably with the TSL, and that learning effects remain stable across sessions^18^. Nonetheless, although in Experiment 1 the left DLPFC conditions were slower than the right DLPFC conditions, tDCS and TMS did not have any effect on TSL.

The absence of stimulation effects may suggest that the DLPFC is not involved in implicit task sequence learning. However, both imaging and patients studies indicate that the DLPFC is involved in implicit sequence learning^23, 47–49^. Since there were also no stimulation effects on task switching performance, which is known to engage the DLPFC^24–26, 50^, a preferable explanation is that the DLPFC was not sufficiently stimulated. Indeed, both theoretical and empirical evidence suggests that the outcome of tDCS and TMS is the sum of the neural activation produced by stimulation and the one produced by the concomitant task^51, 52^. Critically, when this outcome is sub-optimal or supra-optimal the effects are not detectable. Thus, the stimulation protocols used here may have effects on several tasks, as shown by previous studies, but they are not sufficient to affect task sequence learning.

For Experiment 1, another explanation is that the stimulation duration may have been too long. In fact, a previous study has shown that anodal tDCS delivered for 26 minutes can have reverse effects on cortical excitability^53^. Hence, since in the present study tDCS was delivered for 30 minutes, the effects may have been washed out. However, in contrast, a recent meta-analysis revealed that the effects of anodal DLPFC tDCS on cognitive tasks are small and independent of factors such as stimulation duration and intensity^1^. Therefore, it is unlikely that the parameters chosen for tDCS produced the null results.

For Experiment 2, another explanation is that cTBS can induce both inhibitory and excitatory effects. This is particularly true for 50 Hz cTBS^54^. However, a recent study compared the 50 Hz and 30 Hz cTBS protocols and showed that in contrast to the 50 Hz cTBS protocol, the 30 Hz protocol had large and consistent inhibitory effects^55^. As we used this latter protocol in the present study, it is unlikely that the cTBS protocol was the cause of the null results.

Although we do not consider these alternative explanations as likely reasons for the null-effects, they demonstrate that systematic investigations of the parameters of different stimulation protocols and their potential interaction with subtle differences in the cognitive tasks are necessary. Together with our previous study, in which we compared online and offline transcranial DLPFC stimulation with tDCS in a bimanual version of the task sequence learning paradigm, the present study contributes to such a research strategy. Further directions should include different stimulation protocols, stimulation sites, and stimulation techniques, as well as the implementation of repeated stimulation regimens.

In conclusion, a growing number of studies shows that the effects of both tDCS and TMS are not as robust and reliable as originally thought^56–60^. The findings of the present study add to this literature by indicating that established tDCS and TMS protocols do not influence implicit task sequence learning and consolidation.

## Method

### Experiment 1

#### Participants and design

Eighty-nine right-handed participants in this study were assigned to one of the six experimental conditions. All participants gave written informed consent before the experiment. None of them reported psychiatric or neurologic disorders. Participants were blind to the experimental conditions. Two participants were excluded because they had an accuracy below 80% in blocks in which the sequences were embedded (i.e., blocks 5–12), three participants were excluded because the laptop used for testing crashed, and one participant was excluded because the sequences showed in the two sessions were different. The final sample was 83 participants: 12 participants for anodal left DLPFC, 14 for anodal right DLPFC, 13 for cathodal left DLPFC, 13 for cathodal right DLPFC, 16 for sham left DLPFC, and 15 for sham right DLPFC (46 woman, 37 man, mean age = 24, *SD* = 7). Stimulation type (anodal tDCS vs. cathodal tDCS vs. sham) and hemisphere (left DLPFC vs. right DLPFC) were manipulated between subjects and blocks were manipulated within subject, resulting in a mixed design. The study was approved by the Ethical Committee of the Canton Bern, and all the methods were performed in accordance with the Declaration of Helsinki.

#### Materials

The TSL paradigm was adopted from Weiermann *et al*.^13, 27^ ^, cf.^ ^6^. Participants were presented with two kinds of stimuli: digits (1, 2, 3, 4, 6, 7, 8, and 9) or letters (a, e, i, u, c, n, r, and s). The stimuli were presented on the center of a black screen in 32-point Arial font in either green or red color.

tDCS was delivered with a DC stimulator plus (neuroConn, Ilmenau, Germany) connected to two rectangular 5 × 7 cm (i.e., 35 cm^2^) rubber electrodes. The electrodes were inserted into sponges soaked with saline solution to decrease impedance. The sponges were attached to the participants scalp by two rubber straps.

The active electrode was placed above positions F3 or F4 of the 10–20 electroencephalography (EEG) system^61^. The return electrode was placed contralateral to the active electrode above the supraorbital region. Current was delivered at 1 milliampere (mA) for 30 minutes. In the sham conditions current was delivered only for 30 seconds. This procedure gives the same skin sensations than actual tDCS but it does not influence neurons^62^. At the beginning of tDCS all participants described skin itching under the electrodes but no other sensation or adverse effect.

#### Procedure

**Figure 5:**
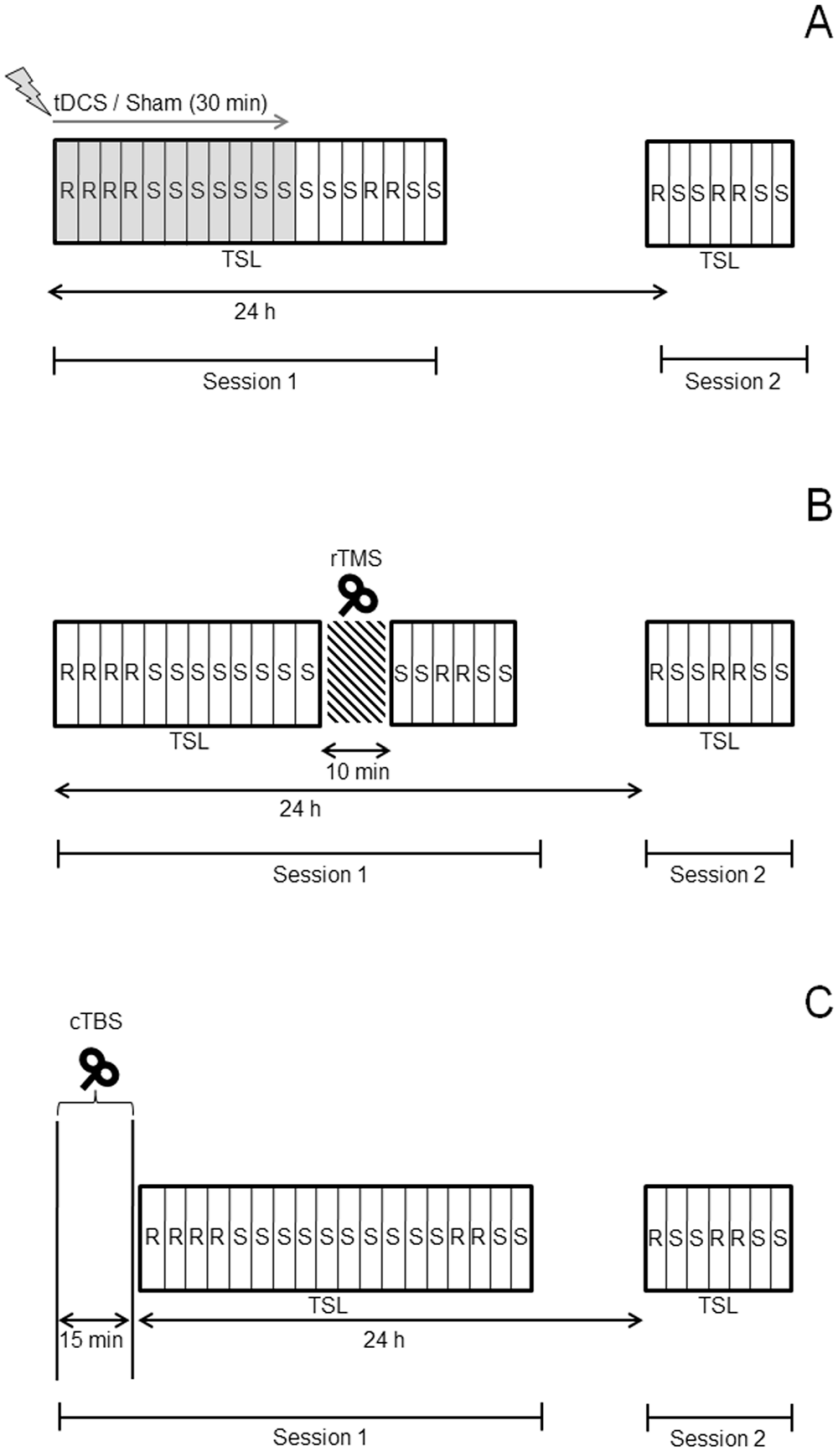
Experimental procedures used in the study, separately for Experiment 1 (**A**) and 2 (**B** and **C**). “R” indicates a pseudorandom block; “S” indicates a sequenced block. The blocks colored in grey indicate the application of tDCS.

**Figure 6:**
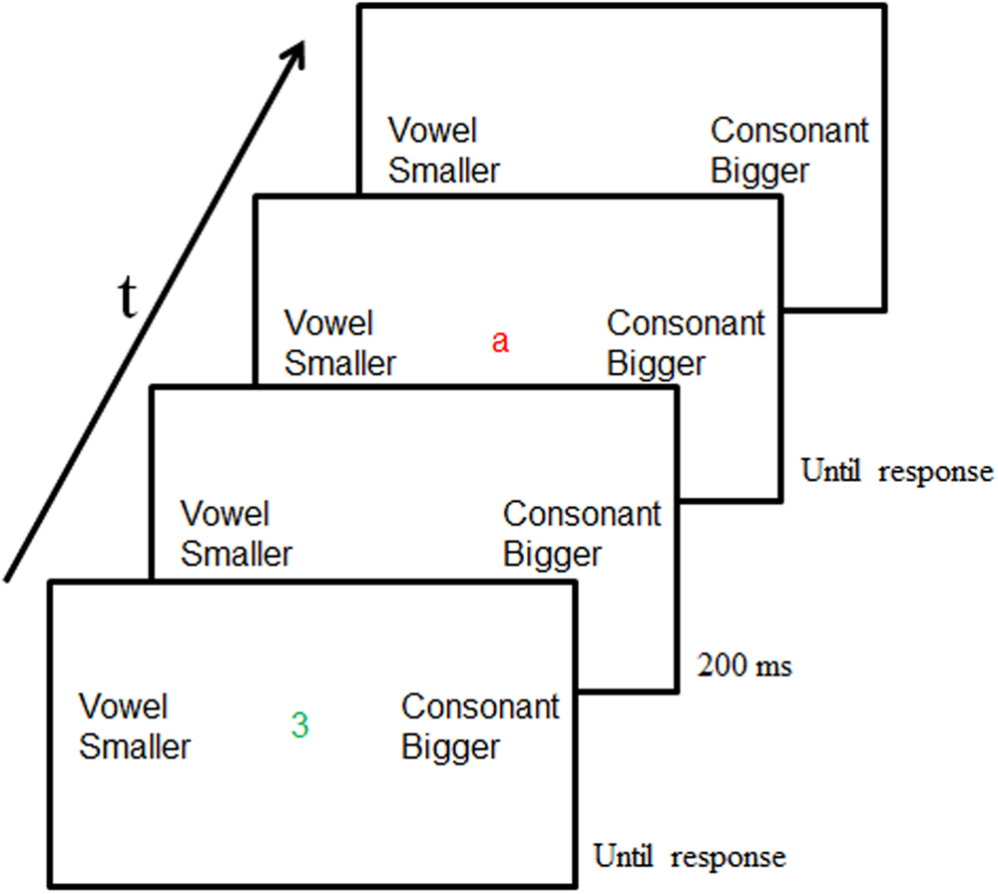
Example of two trials of the task sequence learning paradigm.

Figure 5A depicts the procedure. Participants were tested individually in two sessions separated by 24 hours. In Session 1, tDCS was set up and written instructions were given to the participants. They were informed that they would conduct a reaction time task in which they had to make digit decisions or letter decisions. The digit decision consisted of deciding whether a digit was smaller (1, 2, 3, 4) or bigger than five (6, 7, 8, 9). The letter decision consisted of deciding whether a letter was a vowel (a, e, i, u) or a consonant (c, n, r, s). Hence, the stimuli determined the task type (i.e., digit and letter task). Additionally, the color of the stimuli determined the response mapping: green was compatible and red was incompatible response mapping. Compatible response mapping consisted in pressing keyboard button “N” with the right index finger for digits smaller than five and vowels, and button “M” with the right middle finger for digits bigger than five and consonants. Incompatible response mapping was the opposite. Thus, keyboard button “N” with the right index finger for digits bigger than five and consonants, and button “M” with the right middle finger for digits smaller than five and vowels. Compatible response mapping was indicated by fixed instructional reminders in white color and in 26-point Arial font on the left and the right of the stimuli. Figure 6 depicts two trials of the task. Participants were instructed to respond as fast and precise as possible and were not informed about the presence of a repeating sequence. After ensuring that participants understood the instructions, tDCS was given for 30 s and participants started the task. Sixteen sequences of task-response mapping combinations, each of which consisted of the four possible trial-to-trial relations (task: repeated vs. switched, and response mapping: repeated vs. switched), were created accordingly to the method of Weiermann *et al*.^13^. Each participant was exposed to one sequence drawn from this pool.

Session 1 consisted of 18 blocks. Blocks 1–4 were practice blocks in which a pseudorandom order of task-response mapping combinations was presented. In blocks 5–14 an eight elements sequence of task-response mapping combinations was embedded, making these blocks sequenced blocks. In blocks 15 and 16 the sequenced order was switched to pseudorandom. In blocks 17 and 18 the sequenced order was re-established. In each sequenced block the sequence was repeated 13 times, for a total of 104 trials. On each trial, a digit or a letter in green or red was presented on the center of the screen. The trial ended when the participant pressed one of the two response buttons (i.e., keyboard button “N” or keyboard button “M”) with the index or middle finger of the right hand. The inter-stimulus interval was 200 milliseconds (ms).

Session 2 consisted of seven blocks. After a first pseudorandom practice block, two sequenced, two pseudorandom, and two sequenced blocks were shown. The whole procedure was run using E-Prime version 1.2 (Psychology Software Tools, Pittsburgh, PA).

#### Data analysis

The first trial of each block, trials in which an error was committed, trials after an error, and trials with a RT below 100 ms were excluded. For all statistical analysis an alpha value of .05 was used. Effect sizes are indicated in partial η^2^. All *t*-tests are two-tailed.

### Experiment 2

#### Participants and design

Forty-nine right-handed participants were assigned to one of the three experimental conditions. All participants gave written informed consent before the experiment. All of them conformed to the criteria for TMS application^63^. Participants were blind to the experimental conditions. One participant was excluded because of accuracy below 80% in blocks 5–12. The final sample was 48 participants: 16 for rTMS left DLPFC, 16 for cTBS right DLPFC, and 16 for sham (40 females, eight males, mean age = 23, *SD* = 4). The design was a mixed one, with stimulation type (rTMS left DLPFC vs. cTBS right DLPFC vs. sham) manipulated between subjects and blocks manipulated within subject. The study was approved by the Ethical Committee of the Canton Bern, and all the methods were performed in accordance with the Declaration of Helsinki.

#### Materials

The TSL paradigm was the same as in Experiment 1.

For both rTMS and cTBS a MagPro × 100 (Magventure, Danmark) connected to a figure-of-eight-coil was used (MC-B70 model, Magventure, Danmark). During stimulation, the coil was placed over position F3 or F4 of the 10–20 EEG system^64^. The coil was held tangentially to the scalp with the handle positioned 45° with participants’ sagittal line.

The rTMS protocol consisted of one magnetic pulse at one Hz (i.e., every second) for 10 minutes for a total of 600 pulses. rTMS was given at 90% of participants’ resting motor threshold (RMT). The RMT was identified above the right motor cortex (M1) as the minimum stimulator intensity sufficient to evoke twitches of participants’ relaxed small hand muscles.

The cTBS protocol consisted of 801 pulses delivered in 44 seconds (s). The pulses were divided in bursts. Each burst was composed by three pulses at 30 Hz. Between each burst there were 100 ms. cTBS was given at 80% of participants’ RMT. The RMT was identified above the right M1 as for rTMS.

#### Procedure

As rTMS effects on cortical excitability are acute and short compared to the moderate and long effects of cTBS^65^, the two experimental procedures differed slightly. Figure 5B and C depict the procedures used for rTMS of the left DLPFC and cTBS of the right DLPFC, respectively. rTMS on the left DLPFC was delivered before the blocks used to measure sequence-specific learning (i.e., blocks 13–18). Fifteen minutes after the first cTBS application another cTBS application was delivered to make sure that the right DLPFC was sufficiently stimulated before the start of the TSL^66^. For sham stimulation a coil equipped with a magnetic shield was used^67^.

#### Data analysis

The same data analysis strategy was used as in Experiment 1.

## Electronic supplementary material

Supplementary Information
