# Supplementary material for: No effects of transcranial DLPFC stimulation on implicit task sequence learning and consolidation
Source: Sci Rep. 2017 Aug 29;7:9649. doi: 10.1038/s41598-017-10128-0 (PMC5575284; doi:10.1038/s41598-017-10128-0)
Supplement: Supplementary file 1 — Supplementary Information [file 41598_2017_10128_MOESM1_ESM.docx]

No effects of transcranial DLPFC stimulation on implicit task sequence learning and consolidation

Branislav Savic, Dario Cazzoli, René Müri, & Beat Meier

University of Bern

Switzerland

Supplementary Materials


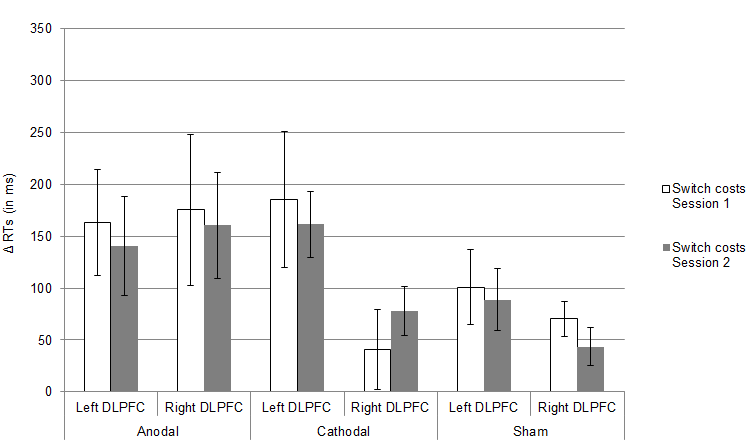
**Supplementary materials Figure S1**:

Fig. S1 Switch costs across sessions, type of stimulation (anodal, cathodal, and sham), and hemisphere (left and right) for Experiment 1. The respective ANOVA showed no significant effect (*ps* > 0.08) indicating that type of stimulation and hemisphere stimulated did not influence performance (see main text).


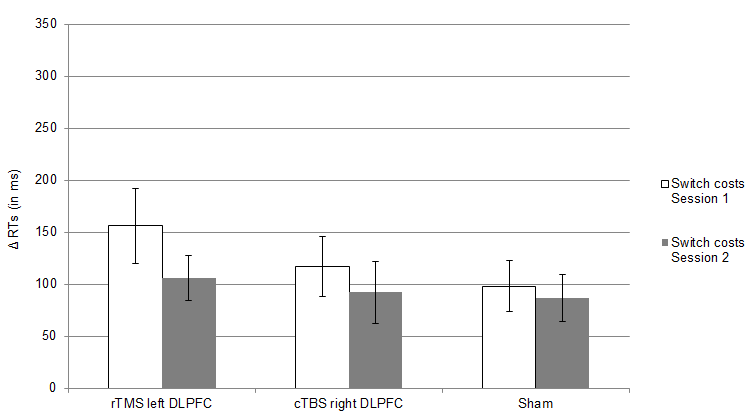
**Supplementary materials Figure S2**:

Fig. S2 Switch costs across sessions and type of stimulation (rTMS left DLPFC vs. cTBS right DLPFC vs. sham) for Experiment 2. The respective ANOVA showed no significant effect (ps > 0.14) indicating that type of stimulation did not influence performance (see main text).
